# Effectiveness and cost-effectiveness of a personalised self-management intervention for living with long COVID: protocol for the LISTEN randomised controlled trial

**DOI:** 10.1186/s13063-023-07090-w

**Published:** 2023-02-01

**Authors:** Claire Potter, Fiona Leggat, Rachel Lowe, Philip Pallmann, Muhammad Riaz, Christy Barlow, Adrian Edwards, Aloysius Niroshan Siriwardena, Nick Sevdalis, Bernadette Sewell, Jackie McRae, Jessica Fish, Maria Ines de Sousa de Abreu, Fiona Jones, Monica Busse

**Affiliations:** 1 Centre for Trials Research, School of Medicine, Cardiff University, Cardiff, Wales UK; 2 Population Health Research Institute, St George’s University of London, London, England, UK; 3 Faculty of Health, Social Care and Education, Kingston University, London, London, England, UK; 4 PRIME Centre Wales, Division of Population Medicine, School of Medicine, Cardiff University, Cardiff, Wales UK; 5 Wales COVID-19 Evidence Centre, Cardiff, Wales UK; 6 Community and Health Research Unit, University of Lincoln, Lincoln, UK; 7 Centre for Implementation Science, King’s College London, London, UK; 8 Swansea Centre for Health Economics, Swansea University, Swansea, UK; 9 Department of Clinical Neuropsychology & Clinical Health Psychology, St George’s University Hospitals NHS Foundation Trust and Mental Health & Wellbeing, School of Health and Wellbeing, University of Glasgow, Glasgow, UK; 10 East Sussex Healthcare NHS Trust Crisis Response Service, St. Annes House, St Leonards-on-Sea, East Sussex UK; 11 Bridges Self-Management, London, England, UK

**Keywords:** Long COVID, Self-management, Rehabilitation, Participation, Process evaluation, Implementation

## Abstract

**Background:**

Individuals living with long COVID experience multiple, interacting and fluctuating symptoms which can have a dramatic impact on daily living. The aim of the Long Covid Personalised Self-managemenT support EvaluatioN (LISTEN) trial is to evaluate effects of the LISTEN co-designed self-management support intervention for non-hospitalised people living with long COVID on participation in routine activities, social participation, emotional well-being, quality of life, fatigue, and self-efficacy. Cost-effectiveness will also be evaluated, and a detailed process evaluation carried out to understand how LISTEN is implemented.

**Methods:**

The study is a pragmatic randomised effectiveness and cost-effectiveness trial in which a total of 558 non-hospitalised people with long COVID will be randomised to either the LISTEN intervention or usual care. Recruitment strategies have been developed with input from the LISTEN Patient and Public Involvement and Engagement (PPIE) advisory group and a social enterprise, Diversity and Ability, to ensure inclusivity. Eligible participants can self-refer into the trial via a website or be referred by long COVID services. All participants complete a range of self-reported outcome measures, online, at baseline, 6 weeks, and 3 months post randomisation (the trial primary end point). Those randomised to the LISTEN intervention are offered up to six one-to-one sessions with LISTEN-trained intervention practitioners and given a co-designed digital resource and paper-based book. A detailed process evaluation will be conducted alongside the trial to inform implementation approaches should the LISTEN intervention be found effective and cost-effective.

**Discussion:**

The LISTEN trial is evaluating a co-designed, personalised self-management support intervention (the LISTEN intervention) for non-hospitalised people living with long COVID. The design has incorporated extensive strategies to minimise participant burden and maximise access. Whilst the duration of follow-up is limited, all participants are approached to consent for long-term follow-up (subject to additional funding being secured).

**Trial registration:**

LISTEN ISRCTN36407216. Registered on 27/01/2022.

**Supplementary Information:**

The online version contains supplementary material available at 10.1186/s13063-023-07090-w.

## Background

In October 2021, the World Health Organization formally defined post-COVID-19 syndrome as an episodic condition with multiple clusters of symptoms [1]. Collectively termed by the community with lived experience as ‘long COVID’ [2], the condition refers to the complex illness and symptoms which persist after an individual contracts the SARS-CoV2 virus. Whilst over 203 symptoms across 10 organ systems have been identified, common symptoms include fatigue, muscle, or joint pain, altered smell and/or taste, breathlessness, cognitive difficulties, and sleep disorders [3, 4]

In the UK, an estimated 2 million people are living with long COVID symptoms, with around 1.7 million having experienced symptoms for at least 12 weeks [5]. Long COVID is adversely affecting everyday lives of 1.5 million people, with many individuals not returning to work by 6 months [3, 5]. Although long COVID services have been set up in many parts of the UK, including NHS long COVID clinics across England and Wales, these have been inaccessible for some, and highly variable in healthcare provision [6]. Whilst NICE guidelines recommend that individuals with long COVID have access to self-management support, to date, effective interventions are lacking.

Proponents of self-management support interventions emphasise the need for contextualisation to (1) specific challenges and complexity of the condition; (2) understanding the setting (community healthcare); (3) training which addresses knowledge, skills and attitudes required by practitioners [7, 8]; and (4) the adoption of specific language and techniques to support key self-management skills such as problem-solving, reflection and personalised goal setting [9].

The Long Covid Personalised Self-managemenT support EvaluatioN (LISTEN) intervention is a co-designed personalised self-management support intervention [10]. It draws on evidence from Bridges Self-management and is informed by self-efficacy as the most successful foundation for self-management programmes [9, 11, 12]. In this approach, therapeutic interactions become less directive and more collaborative, facilitating individuals’ problem-solving skills, goal mastery and building self-efficacy [13]. The intervention incorporates growing evidence that people living with long COVID experience diverse and fluctuating symptoms which can be triggered and made worse by physical tasks and exertion as well as emotional, social, and cognitive factors. In addition, that people living with long COVID can face disbelief and stigma, including from health professionals and need to feel heard and their experiences validated [2, 14]. Throughout this article, when we describe the ‘LISTEN intervention’ we are referring to the aforementioned co-designed personalised self-management support intervention [10].

## Objective

The objective of the LISTEN two-arm randomised controlled trial with embedded process evaluation is to evaluate the effectiveness of the LISTEN personalised self-management support intervention for non-hospitalised people living with long COVID on participation in routine activities, social participation, emotional well-being, quality of life, fatigue and self-efficacy when compared to usual NHS care. Cost-effectiveness will also be evaluated. Intervention acceptability and feasibility will be measured as part of the embedded mixed methods process evaluation, which will provide a detailed analysis of implementation enablers and barriers to adoption and sustainability beyond the study timeline. This work will inform and deliver an implementation support package (for example training programme for rehabilitation teams, web platform, training manuals etc.) ready for national scale-up and implementation within the NHS by the end of the study.

## Methods

### Trial design and study setting

**Fig. 1:**
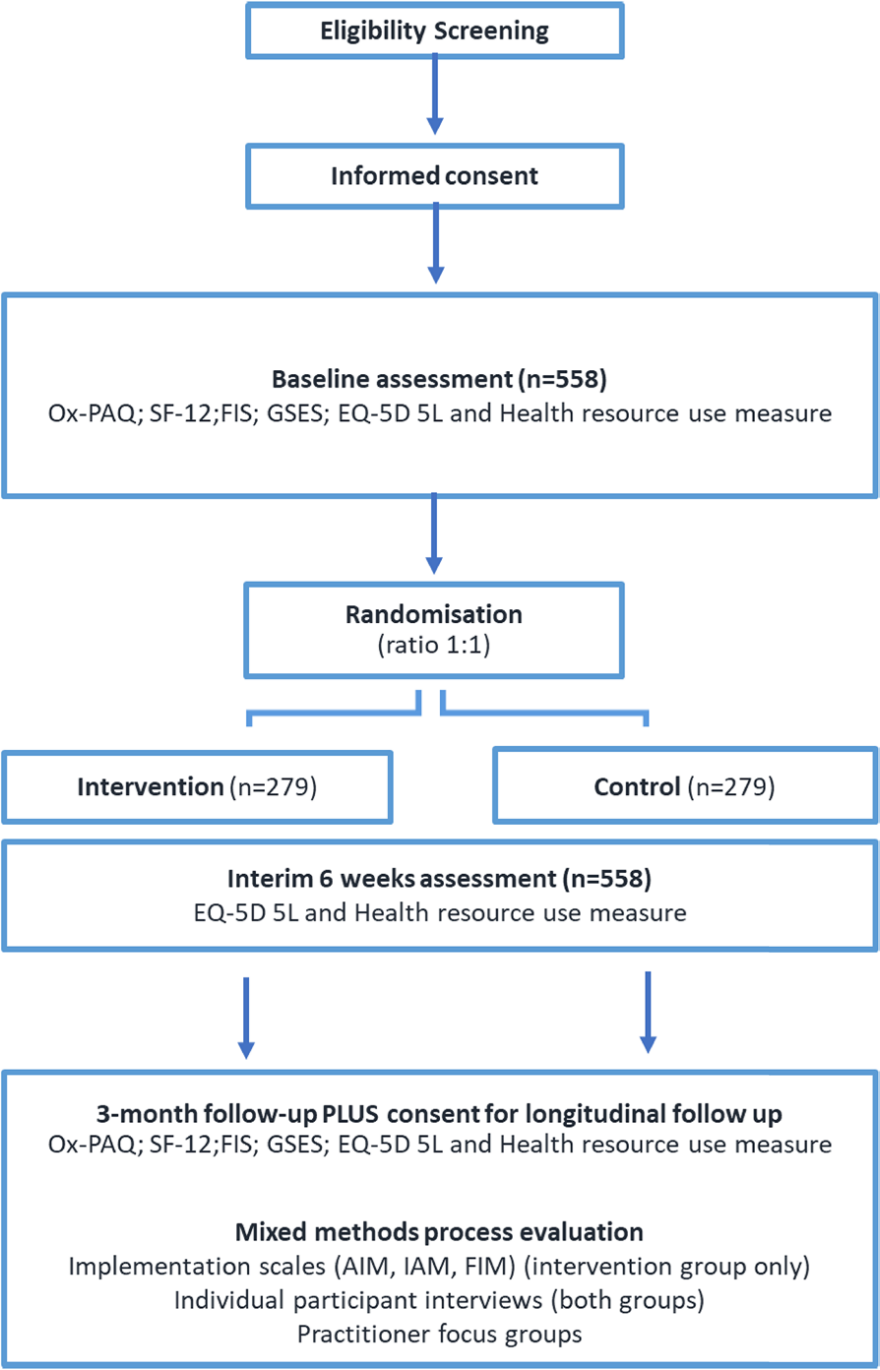
Participant flow diagram

This is a two-arm individually randomised controlled trial for people living with long COVID, with an embedded cost-effectiveness and mixed-methods process evaluation (see Fig. 1; participant flow diagram). The trial methods were developed and written in line with the SPIRIT reporting guidelines [15].

Following informed consent and completion of baseline outcome measures, 558 participants will be randomised to either continue receiving usual care or receive the LISTEN intervention for a 3-month period. Recruitment is anticipated to last 9 months, with each participant taking part in the trial for 3 months (see Table 1; SPIRIT schedule of events) and the last follow-up due 12 months after start of recruitment. Participants will be recruited from England and Wales through a number of methods including self-referral to enhance inclusivity. Primary and Secondary care centres within England have been set up as individual sites and Wales has been set-up as one site covering all seven Health Boards.

**Table 1:**
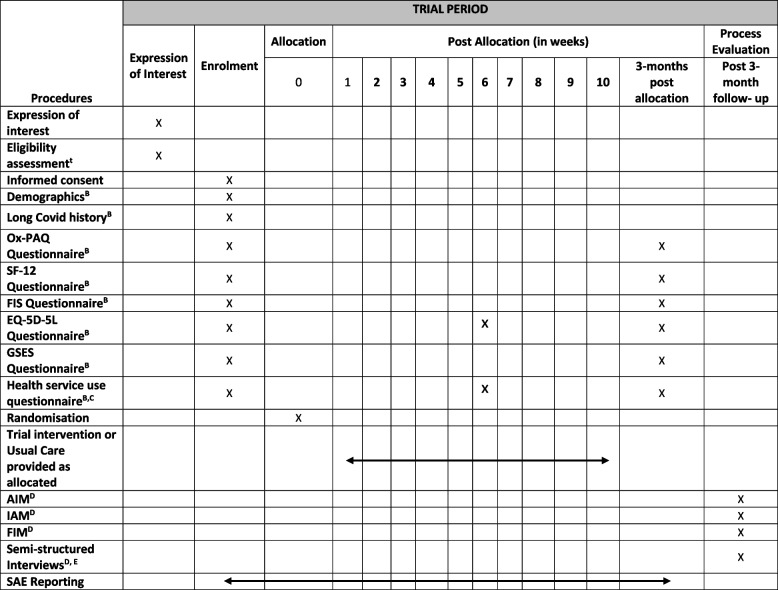
SPIRIT schedule of events

^A^A random selection of participants will be phoned by the central CTR team for an eligibility review

^B^If participants are unable/unwilling to complete the questionnaires online, a paper/hard copy can be sent in the post and the forms completed over the telephone with a member of the central CTR team

^C^A diary will be available to download to help participants record their appointments to complete the health service use questionnaire

^D^Process evaluation questionnaires will be completed only by participants in the LISTEN intervention group. Interviews will be conducted with a subset of participants and will include both those who received the LISTEN intervention and usual care

^E^Process evaluation interviews and focus groups will also be conducted with a subset of staff members involved in LISTEN delivery

Those who are eligible and consent to participating in the study will complete a series of questionnaires at baseline and then repeat a selection at 6 weeks and 3 months post randomisation (see supplementary materials). Participants and practitioners will be invited to consent to taking part in process evaluation which will involve implementation scales, interviews, and focus groups.

### Eligibility criteria

Eligible participants are people who are aged 18 years or older, are an English or Welsh speaker or have access to someone who can act as a translator. They must have experienced at least one long COVID symptom [4] for 12 weeks or longer and additionally meet at least one of the following criteria: (i) positive SARS-CoV-2 PCR or antigen test (positive COVID-19 test) during the acute phase of illness, (ii) positive SARS-CoV-2 antibody test (positive COVID-19 antibody test) at any time point in the absence of SARS-CoV-2 (COVID-19) vaccination history, (iii) loss of sense of smell or taste during the acute phase in the absence of any other identified cause, (iv) symptoms consistent with SARS-CoV-2 (COVID-19) infection during the acute phase and high prevalence of COVID-19 at time and location of onset, (v) at least one symptom consistent with SARS-CoV-2 (COVID-19) infection during the acute phase and close contact of a confirmed case of COVID-19 around the time of onset. Potential participants must have consulted with their GP to rule out serious complications or the need for further investigation in relation to persistent symptoms following COVID-19 infection.

Individuals who have a palliative condition, or are receiving end-of-life care, were hospitalised during the acute phase of COVID-19 for treatment of their COVID symptoms or are actively participating in another long COVID intervention trial will not be eligible to take part in LISTEN. Individuals who have participated in the LISTEN co-design activities will also not be eligible for the trial.

### Recruitment

Potential participants will be identified by the following methods:

1. LISTEN sites will identify participants from their long-COVID service records and waiting lists
2. GP practices set up as participant identification centres (PICs) will send out invitation letters to potentially eligible participants via the Docmail system. Docmail is a secure hybrid mail solution that enables account holders to create and despatch mailings.
3. Publicity and social media advertisement to promote self-referrals via the LISTEN website [16].

All participant outward-facing communications (including audio and filmed materials) about the project have been reviewed by our Inclusion Advisors (Diversity and Ability Social Enterprise [17], and members of our PPIE group to ensure they are accessible, inclusive and representative of a range of populations.

### Expression of interest and consent

Potential participants identified by LISTEN sites or responding to advertisement will be directed to the study website [16]. The website contains information and videos explaining the design of the LISTEN intervention, a map detailing where current LISTEN sites are based and an audio and written version of the participant information sheet. Interested people are invited to complete an expression of interest form within the website. This form collects contact details, GP details and eligibility information. Only the central trials team will have access, via individual logins, to access the information provided within the expression of interest form.

The central trial team will check through all expressions of interest received through the website to assess for eligibility. Potential participants within England are only eligible if they are within the catchment area of a LISTEN site; this is confirmed by their GP postcode. However, one non-NHS site covering England may be opened to cover participants in more remote locations. Wales has been opened as a single site covering all health boards which allows participants from any postcode within Wales to participate. Personalised emails will be sent in response to all expressions of interest to thank them for their interest and let them know from the information they have provided whether they are eligible to participate in the study or not. Twenty per cent of potential participants deemed eligible, will be telephoned to check eligibility answers. Eligible participants who can be allocated to a LISTEN site will be added to the LISTEN REDCap database and will be sent a link via email to complete the electronic consent form.

If those interested in participating are unable or unwilling to use the internet, local advertisements will also contain a central phone number for potential participants to contact the central trial research team, who can provide assistance over the telephone to complete the expression of interest and consent forms. Alternatively, participants identified by a LISTEN site can complete consent over the telephone or in person with a delegated member of the LISTEN site team.

### Baseline assessments

Once a participant has consented to participate in the study, they will be sent a series of emails with a link to self-complete the required outcome measures. Based on from the LISTEN PPIE group, advice to mitigate the impact of fatigue and minimise burden, the questionnaires will be sent in three batches and participants will be informed that the questionnaires do not need to be completed immediately, and breaks should be taken if needed. The link will take participants directly to the questionnaires and these data are automatically saved within the LISTEN database. The central trial research team will be notified when all baseline questionnaires have been completed. For participants who are unable or unwilling to use the internet, baseline assessments will be undertaken by the trial team via telephone.

### Randomisation method and implementation

Participants will be individually allocated to the intervention or usual care arm using simple randomisation stratified by site. This will be implemented via the LISTEN secure remote web-database on the completion of baseline assessments. The randomisation sequences will be generated in Stata 17 using permuted blocks of randomly varying sizes between 2 and 10. All forms and questionnaires have in-built validations for mandatory responses and will be reviewed by a member of the central trials research team to ensure completeness before initiates randomisation. Email notifications will be sent to the participant and their allocated site informing them which group the participant has been randomised to.

## Interventions

### LISTEN intervention

**Fig. 2:**
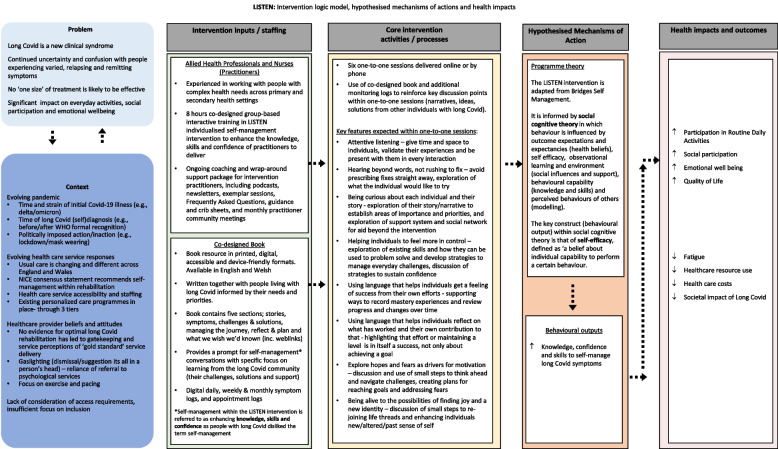
LISTEN intervention logic model

Participants allocated to the LISTEN intervention will received a personalised self-management support package. To avoid contamination, participants allocated to this group will be asked to not access local rehabilitation services until after the 3-month follow up is complete. The trial intervention will comprise two components, (a) up to six remotely delivered (via a secure web video conferencing system or telephone) one-to-one personalised self-management support sessions (over a 10-week period, each maximum of 1 h) and (b) access to print and web-based resources. These resources primarily refer to a LISTEN book, co-designed by people with lived experience of long COVID and sent to participants for use within the one-to-one sessions [10]. The book includes narratives of individuals with long COVID, their challenges and problem-solving ideas for navigating everyday life. Use of personal communities, including peer-support groups, and specific online information sources will also be encouraged. The intervention is underpinned by social cognitive theory, and specifically self-efficacy theory [18]. Key sources of self-efficacy, including mastery and vicarious experiences are integral to the intervention, as proposed mediators of change. Self-efficacy is also an anticipated outcome (see Fig. 2; intervention logic model). Participants are expected to complete a minimum of four out of the six sessions but if the participant deems to have taken what they can from the sessions they can complete fewer.

Participants allocated to the intervention arm will receive one-to-one support sessions from NHS practitioners specifically trained to support people with long COVID [10]. Practitioners will primarily be nursing and allied health professionals including physiotherapists, occupational therapists, nurses, and clinical psychologists. Before delivering one-to-one sessions, practitioners will have attended 8 h of training in the form of four, 2-h online workshops adapted from Bridges self-management during the intervention co-design process [10]. During workshops, practitioners will be trained in nine key skills, forming key components included in the fidelity checklist of the LISTEN intervention (see Fig. 2). Practitioners’ use of these skills (e.g. attentive listening, exploring needs and priorities, and exploring how to use language that helps individuals feel their progress and successes), aims to enhance participants’ knowledge, skills, and confidence in self-managing everyday activities with long COVID. The training will be evaluated via an online Likert scale survey to assess practitioners’ knowledge and confidence in using the key skills prior to intervention delivery.

To supplement the training and maintain intervention fidelity, practitioners will be given access to a wrap-around support package when delivering the intervention. Hosted electronically using MS Teams, it is expected that this platform will support practitioners in all aspects of intervention delivery and respond to collective needs identified through the training evaluation data and ongoing practitioner communication. The platform will contain supporting video and audio files, documents, such as frequently asked questions and crib sheets, and recordings from any interactive ‘top-up’ training events hosted.

### Fidelity of LISTEN intervention delivery

To assess intervention fidelity, 10% of one-to-one remote sessions delivered will be independently analysed. Sessions will be recorded via Zoom or MS Teams depending on participants’ preferences and reviewed against nine predefined skills, using a fidelity checklist, featured within the practitioner training. The checklist will be used for collective analyses across a participants’ one-to-one sessions, as opposed to independent analyses on each one of the six sessions. Practitioners may be asked to record any participant’s six sessions beyond that of their second participant. Reflections from the practitioners delivering the intervention will be captured through online notes pages. Structured using the key skills of the intervention, notes will be completed after each participant session, totalling six entries, to encourage the sustainment of intervention delivery. Participants will be made aware if their sessions will be recorded, and each recording will be stored in a confidential space only accessible by the trial team, and the specific practitioner.

### Usual care

All participants randomised to usual care will continue to receive the NHS long COVID services currently available their respective regions. In July 2022, NHS England commissioned updated guidance for post covid services, providing referral routes and criteria for post Covid services, including self-management support, multidisciplinary rehabilitation, and specialist referral. However, provision and access to services across the UK is variable, ranging from referral to one of 90 specialist long COVID clinics, to use of a digital platforms such as Your COVID recovery website or Living with Covid Recovery [19, 20]. The LISTEN team will signpost participants to information about local services as required. For some participants, usual care may involve actively receiving their local long COVID services, being on a waiting list for a service, or exploring the options available to them through their GP. Being in the usual care arm of the LISTEN study will not fast track participants to receive long COVID services. We will assess usual care within the trial regions during site set-up and during the process evaluation with site clinical practitioners towards the end of the trial. Given the potential for contamination, we will also gather detailed records of usual care (captured from a health services resource questionnaire) in those randomised to the comparator arm as part of our process evaluation.

### Follow-up

Six weeks after randomisation, all participants will receive an email requesting for them to complete a sub-set of the questionnaires primarily focussed on health services use, answering all questions relating to the last 6 weeks. Three months after randomisation, all participants will receive a series of emails requesting the completion of the full set of questionnaires completed at baseline. Participants can contact the central trials research team for assistance in completing these questionnaires over the phone.

### Process evaluation

**Fig. 3:**
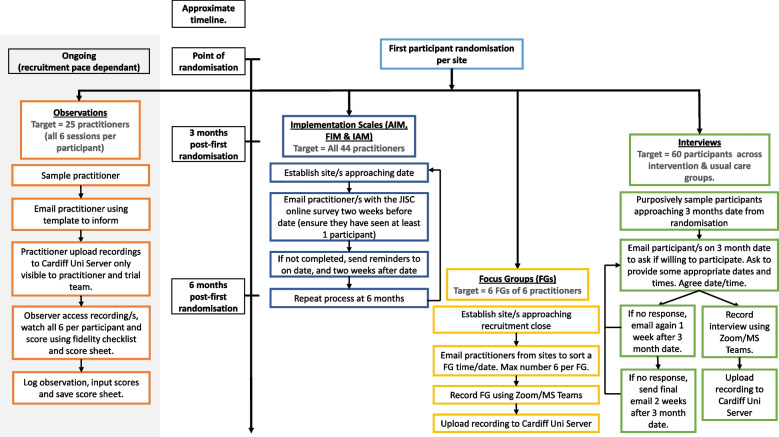
LISTEN process evaluation flow chart

A theory-driven, embedded process evaluation which has been designed in accordance with the MRC framework [21] for evaluation of complex interventions will be conducted. This will utilise multiple methods, including validated surveys, interviews, and focus groups (see Fig. 3).

To guide the collection of implementation data, the Proctor implementation outcome taxonomy [22] will be applied, offering the current gold standard in conceptually articulating different aspects of implementation to be assessed. Of this taxonomy, we will assess the acceptability, feasibility, and appropriateness of LISTEN as perceived by the service users who have completed the LISTEN intervention (3 months after randomisation), and practitioners (3 and 6 months after the first randomisation at their site), thus facilitating the assessment of implementation of LISTEN at both earlier and more advanced stages. The brief validated scales (4 items each; 12 implementation items in total; all scored on 5-point Likert scales) that will be used include the Acceptability of Intervention Measure (AIM), the Intervention Appropriateness Measure (IAM), and Feasibility of Intervention Measure (FIM) [23]. All participants who complete the LISTEN intervention will be asked to complete the implementation measures and all practitioners delivering the intervention will be invited to also complete the same three scales.

Semi-structured interviews will be conducted with a sub-set of long COVID participants (up to *n*=60). All interviews will be carried out remotely after the primary outcome data collection time point (3 months). Interviews with participants who received the LISTEN intervention will explore experiences of engaging with LISTEN, the perceived acceptability, feasibility, and appropriateness for them as an intervention to address their needs, experiences of the rehabilitation services through which the LISTEN intervention will be offered if found to be effective and cost-effective, challenges faced in accessing or receiving LISTEN, and how they overcame such challenges and completed the LISTEN trial. Interviews with those who received usual care will focus on the content and experiences of the NHS long COVID care services received. Participants will be sampled purposively with the aim of obtaining a maximum-variation sample that reflects common UK population characteristics (e.g. gender, age, ethnicity), length of time living with long COVID symptoms, and a spread across different sites and long COVID usual care services.

With this sample it is expected that thematic saturation in what participants report, in relation to LISTEN delivery, will be achieved. The topic guide for the interviews will be designed jointly with our PPIE panel and people living with long COVID who have already informed our study design stage, prior to funding being made available.

Finally, up to six focus groups will be conducted with an average of six participants per group, providing representation across all practitioners delivering LISTEN. These groups will be used to explore, (a) how training was delivered to staff and its acceptability to them, (b) how support was administered and whether staff perceived that they were able to deliver the intervention as intended, (c) any modifications required to assist intervention delivery and (d) any contextual factors within practitioner teams and the wider health service environment that affected intervention delivery in the trial. This will increase understanding of barriers and facilitators and generate insights into how this might enable or prevent sustainability and spread of the LISTEN intervention, thereafter, should it be shown to be effective and cost-effective.

### Internal pilot

An internal pilot will assess site opening and recruitment. The traffic light system (green, amber, red) of progression criteria as proposed by Avery et al [24] will guide decision-making with green resulting in the trial continuing as planned; amber, the trial continuing with changes; red: the trial stops.

### Primary outcome

The primary outcome measure is the routine activities scale domain of the Oxford Participation and Activities Questionnaire (Ox-PAQ) [25]. The Ox-PAQ is a 23-item, fully validated patient-reported outcome measure developed specifically to assess participation and activity in individuals with chronic health problems. Ox-PAQ items have been generated using the World Health Organization (WHO) International Classification of Functioning, Disability and Health (ICF) as a theoretical framework. Participation is reflected across three domains, namely Routine Activities (14 items), Emotional Well-Being (5 items) and Social Engagement (4 items), all of which demonstrate sound psychometric properties in terms of validity, reliability, and sensitivity to change [25].

### Secondary outcomes

i. Emotional Well-Being assessed by the Ox-PAQ [25]
ii. Social Engagement assessed by the Ox-PAQ [25]
iii. The Short Form-12 (SF-12) Health Survey [26], a 12-item, patient-reported survey of patient health, will facilitate examination of physical, social, and emotional domains relevant to health-related quality of life.
iv. The Fatigue Impact Scale [27] (FIS), a 40-item form, will provide an understanding of the impact of fatigue on cognitive, physical, and psychosocial functioning in daily living.
v. The generalised Self-Efficacy Scale [28] (GSES) assesses perceived self-efficacy to predict coping with daily struggles and adaptation after experiencing stressful life events. The GSES with its 10 items and additional context specific questions will allow us to explore the key anticipated mediators of intervention outcome (namely self-efficacy in the context of COVID-19).
vi. EQ-5D-5L will provide Information on multi-attribute utility including its five dimensions of health mobility, self-care, usual activities, pain/discomfort, and anxiety/depression [29]
vii. Client Services Receipt Inventory (CSRI) [30] adapted specifically to capture health care resource use in patients with long COVID.

### Process evaluation measures

i. Perceptions of acceptability and feasibility of trial processes (randomisation, outcomes measures etc.).
ii. Perceptions of acceptability and usability of co-design resources – book and digital.
iii. Perceptions of training and wrap-around support for intervention practitioners.
iv. Perceptions of acceptability and feasibility of one-to-one support sessions for intervention practitioners.
v. Skills required to deliver the intervention and alignment with intervention fidelity.
vi. Participant perceptions of intervention success and the extent to which they match to outcomes measured and outcomes important to those delivering the intervention.
vii. Processes which facilitate and/or act as barriers to implementation, including contextual factors such as organisational, personal, or professional issues.
viii. Factors required to enable sustainability and spread to other NHS sites beyond the project timeline.

### Sample size

The LISTEN study aims to recruit 558 individuals with long COVID. We aim to detect a minimum clinically important effect size of 0.32 [25] between randomised arms in the primary outcome of the Routine Activities domain of the Ox-PAQ with 90% power whilst controlling the two-sided type I error level at 5%. A conventional individually randomised trial would require 414 participants (based on a two-sample *t*-test). However, assuming the intervention would be delivered by 24 community rehabilitation teams, potential clustering in the intervention arm was taken into account. Assuming an intraclass correlation coefficient (ICC) of 0.03, 24 clusters with 10 participants each in the intervention arm and 234 participants in the usual care arm (i.e. a total of 474 participants) are required for 90% power. This was calculated using the method of Moerbeek and Wong [31] as implemented in version 0.7.0 of the R package clusterPower [32]. Assuming 15% loss to follow-up, the overall recruitment target is 558.

### Data collection methods

For the initial self-referral expression of interest form, a publicly available survey has been developed on Jisc Online Surveys tool [33]. This same online survey platform has been used to create the withdrawal of consent form and report a problem form. Links for both forms can be found on the LISTEN website and have been emailed to all consented participants. All data entered by participants are stored securely and confidentially. Only central trial team staff have login access to the survey data on secure Cardiff University servers.

Study data and electronic consent are collected and managed using REDCap electronic data capture tools [34, 35] hosted at Cardiff University. REDCap (Research Electronic Data Capture) is a secure, web-based software platform designed to support data capture for research studies, providing (1) an intuitive interface for validated data capture; (2) audit trails for tracking data manipulation and export procedures; (3) automated export procedures for seamless data downloads to common statistical packages; and (4) procedures for data integration and interoperability with external sources. In REDCap, participants are viewed using a unique trial identifier on the LISTEN database. Personal identifiable information can only be accessed by the trial team and allocated site staff on the database by opening a record. All site staff members will be provided with individual logins once all required documents have been completed.

### Statistical analysis

Participant characteristics and outcome scores at baseline will be summarised descriptively by randomised allocation (usual care or intervention). The primary analysis will use the intention-to-treat population, i.e. participants will be analysed as having received what they were randomised to, regardless of actual level of adherence. To assess intervention effectiveness in terms of the primary outcome, we will fit a linear mixed-effects regression model with the Ox-PAQ Routine Activities scale score (RASS) at 3-month follow-up as dependent variable and the randomisation allocation and baseline RASS as independent variables, and a random site effect. We will present the estimated difference of the RASS at 3-month follow-up between intervention and usual care with a two-sided 95% confidence interval and *p*-value, testing against a two-sided significance level of 5%.

To account for potential additional clustering due to the intervention being delivered by multiple community rehabilitation teams within each site, we will introduce a nested random effect in the intervention arm only, based on the ‘partially clustered’ modelling approach [36, 37]. Similar analyses will be performed for the secondary outcomes.

In secondary analyses, we will add the validated implementation scales as covariates into the model, to assess the impact of implementation perceptions on the outcome measure. We will also adjust the effect of intervention for variables such as age, gender, ethnicity, BMI, index of multiple deprivation, employment status, severity of long COVID at baseline, duration of long COVID, use of health and social care services, and comorbidities. Subgroup analyses will be for type and intensity of the usual care received, and for medication or other therapies participants used to relieve long COVID symptoms.

To investigate the impact of missing data on results, sensitivity analysis for missing observations in outcomes and other independent variables using multiple imputation with the assumption of missingness at random (MAR) will be conducted. We will further conduct sensitivity analyses based on the interventions participants actually received.

A detailed statistical analysis plan (SAP) will be finalised and signed off before any analysis is performed. All analyses will be performed using Stata version 17.

### Health economic evaluation

The base case analysis will take an NHS and Personal Social Services perspective. In addition, we will record patient expenses and loss of productivity to gauge the impact of the intervention on the burden to the patient and society. We will investigate the implementation cost of the intervention (including training, staff, online resources, e.g., hosting and access,) compared to usual care through review of study notes and discussions with the study team. Furthermore, we will collect participant’s healthcare resource use using a Client Services Receipt Inventory, specifically adapted to individuals with long COVID. Health care resource use will be collected in the 3 months before baseline (through patient recall with the option to provide longer-term resource use if patients wish to do so) and at the 3-month follow-up point for both usual care and intervention groups to estimate the impact of the intervention on use of healthcare resources in primary, secondary and social care as well as patient out-of-pocket expenses and ability to undertake paid work.

A *cost-utility analysis* will be undertaken commensurate with the statistical analysis (regarding primary analysis population, handling of missing data) and will calculate the cost per quality-adjusted life-year gained (QALY) gained based on EQ-5D-5L responses at baseline and 3-month follow-up. A cost-consequences analysis will be conducted, and net monetary benefit calculated to weigh up all costs and outcomes of the intervention. Sensitivity and scenario analyses will explore the impact of uncertainty on the results.

### Qualitative analysis

All interview and focus data will be transcribed verbatim. Transcripts will be uploaded in NVivo version 1.0 to enable the initial coding and categorisation of raw data. Descriptive themes will be crafted inductively from core patterns of meaning within the data. Further to these data-driven themes that are developed from interviews with patients and focus groups with providers, the Consolidated Framework for Implementation Research (CFIR) will be applied to interrogate findings from an implementation perspective [38, 39]. The CFIR is a well-established implementation framework which aims to understand factors that impact upon successful implementation, and then address them. Using the CFIR, and in the context of the study, five major determinants of the implementation of LISTEN can be suggested (i) LISTEN itself as an intervention, including its theoretical underpinnings, (ii) The implementation process, (iii) The people involved in designing and implementing LISTEN, (iv) The local context in the long COVID services within the trial (including defining usual care across trial regions), and (v) The wider context of the NHS and the ongoing pandemic. Use of the CFIR during the analysis will enable barriers and drivers of implementation from the perspective of the service users and providers to be mapped onto potential implementation support strategies as they have emerged in the CFIR evidence base [40], and in addition to whichever strategies might emerge from the process evaluation itself.

The process evaluation will be carried out prior to the final statistical analysis to minimise research interpretation being influenced by knowledge of the primary and secondary outcomes. The proposed methods will enable the comparison and collation of multiple data sources with theory and provide a richer understanding of the functioning of the intervention, mechanisms, contextual factors, and implementation processes to support LISTEN beyond this study. A revised logic model for LISTEN will be produced upon completion of the process evaluation to help support subsequent scale-up.

### Data monitoring and audit

A Trial Management Group (TMG) will meet monthly to review study progress and recruitment targets. In addition, there is an external Trial Steering Committee (TSC), with a sub-set of members acting as a Data Monitoring Committee to independently review participant safety. Members of all committees have signed the trial-specific charter. A trial monitoring plan has been developed by the CTR, to determine the intensity and focus of central monitoring activity. LISTEN investigators agree to allow trial-related monitoring, including audits and regulatory inspections, by providing access to source data/documents as required. Findings generated from central monitoring will be shared with the TMG.

### Safety

Participants will be asked to use the Jisc Online Surveys “Report a Problem” link to self-report any relevant medical events during their time on the trial (consent to 3-month follow-up). This link is emailed to all consented participants and is also available on the LISTEN website. We ask for participants to report if they are admitted to hospital for any reason, if their long COVID symptoms get substantially worse or if there is any other incident they feel is relevant to report. This form will ask them for information regarding the onset of the event, detailed description of the event and treatment received. All “Report a Problem” forms submitted will be reviewed by the central trials team for completeness and uploaded to the LISTEN database. The site Principal Investigator will be requested to review the event reported and determine if it meets the trial definition of an adverse event (AE) or a serious adverse event (SAE). If it does, an AE/SAE form will be completed by the site team. LISTEN practitioners can also report AEs or SAEs if this information is provided to them within a LISTEN intervention session.

For the purpose of this trial, only AEs relating to psychological distress and any events meeting the definition of a SAE will be recorded. All SAEs that occur between the time of consent and the 3-month follow-up must be reported immediately to the central trial team and within 24 h of knowledge of the event.

### Ancillary and post trial care

All those allocated to usual care will be sent a printed copy of the LISTEN book following completion of their 3-month follow-up.

### Roles and responsibilities

Kingston University is the Sponsor for the trial. The Sponsor has delegated trial management and main analysis responsibilities to the Centre for Trials Research (CTR) at Cardiff University. Professor Fiona Jones and Professor Monica Busse are co-chief investigators and designed the LISTEN study alongside co-applicants from Cardiff University, Kings College London, University of Lincoln, Swansea University and St George’s University Hospitals NHS Foundation Trust including a public and patient involvement and engagement (PPIE) representative. All activities within this trial will adhere to the CTR’s Standard Operating Procedures (SOPs), including those for data management and protection. The process evaluation data collection and analysis will be conducted by Kingston University and Kings College London. The health economics analysis will be conducted by Swansea University.

## Discussion

Long COVID likely affects around 2 million people in the United Kingdom. This trial will examine the effectiveness and cost-effectiveness of the LISTEN intervention across multiple sites and services in England and Wales. The process evaluation and implementation framework analysis will inform subsequent dissemination, scale-up, spread and how the pathway to impact can be enabled, should the intervention be shown to be effective and cost-effective.

To maximise inclusion, the trial allows for self-referral and various modes of information provision (written/visual), taking consent, data collection and intervention delivery (online or verbal) have been employed. Wales has opened to recruitment under a ‘One Wales’ model facilitating recruitment across Wales regardless of postcode. We are seeking to replicate this reach in England by use of a non-NHS site to accept referrals outside of participating site catchment areas. Extensive PPIE has been employed in the project from the initial co-design of the intervention [10], development of the trial protocol, associated materials and process evaluation through to support with ongoing recruitment strategies and advertising, and latterly dissemination planning.

This study is one of a range of COVID-19 research studies currently funded by NIHR in UK. Other studies will describe the epidemiology and natural history, phenotypes of and within the condition, and the effectiveness of current services available across the UK. Notable among the latter is the LOCOMOTION study evaluating 10 usual care long COVID services in England, Wales and Scotland [41]. The LISTEN trial will assess the effectiveness and cost-effectiveness of the LISTEN intervention over and above existing services provision and will provide a vital piece of the evidence base as to whether services should integrate this intervention as well, and the benefits and costs of doing so. It will inform whether current guidance and policy should be updated to include such self-management support interventions via local ‘usual care’ services.

## Trial status

This manuscript has been drafted according to version 3.0 (29/03/2022) of the trial protocol and in accordance with Standard Protocol Items: Recommendations for Interventional Trials (SPIRIT) 2013 Statement (see Additional file 2 – SPIRIT Checklist). The trial opened to recruitment on 27/05/2022 and recruitment is anticipated to be completed on 28/02/2023.

## Supplementary Information

**Additional file 1: Appendix 1.** Model Informed Consent Form. **Appendix 2.** Model Participant Information Sheet.

**Additional file 2.** SPIRIT Checklist for Trials.

## Data Availability

Trial data will be stored in the LISTEN Research Data Storage space in a secure repository held by Cardiff University. CTR is a signatory of AllTrials and aims to make its research data available wherever possible. Data requests will undergo a review process to ensure that the proposal complies with patient confidentiality, regulatory and ethical approvals and any terms and conditions associated with the data. On study completion, trial data will be made available upon reasonable request study completion and following CTR data sharing policies. Requests to access the data should be made to the ctrdatasamplerequests@cardiff.ac.uk.

## Abbreviations

AE: Adverse event
AIM: Acceptability of Intervention Measure
BMI: Body mass index
CEO: Chief Executive Officer
CFIR: Consolidated Framework for Implementation Research
CSRI: Client Services Receipt Inventory
CTR: Centre for Trials Research
EQ-5D-5L: European Quality of Life-5 Dimension 5 - Level (adult quality of life instrument)
FIS: Fatigue Impact Scale
FIM: Feasibility of Intervention Measure
GP: General practitioner
GSES: Generalised Self-Efficacy Scale
IAM: Intervention Appropriateness Measure
ICC: Intraclass correlation coefficient
ICF: International Classification of Functioning, Disability and Health
LISTEN: Long Covid Personalised self-management support Evaluation
MAR: Missingness at random
MRC: Medical Research Council
NHS: National Health Service
NICE: National Institute for Health and Care Excellence
NIHR: National Institute for Health Research
Ox-PAQ: Oxford Participation and Activities Questionnaire
PCR: Polymerase chain reaction
PIC: Participant identification centre
PPIE: Public and Patient Involvement and Engagement
QALYS: Quality-adjust life years
RASS: Routine activities scale score
R&D: NHS Trust Research and Development Department
REC: Research Ethics Committee
REDCap: Research Electronic Data Capture
SAE: Severe adverse event
SAP: Statistical analysis plan
SF-12: Short Form-12
SOP: Standard Operating Procedures
SPIRIT: Standard Protocol Items: Recommendations for Interventional Trials
TMG: Trial Management Group
TSC: Trial Steering Group
UKECA: United Kingdom Ethics Committee Authority
WHO: World Health Organization
