# Supplementary material for: Effectiveness and cost-effectiveness of a personalised self-management intervention for living with long COVID: protocol for the LISTEN randomised controlled trial
Source: Trials. 2023 Feb 1;24:75. doi: 10.1186/s13063-023-07090-w (PMC9890432; doi:10.1186/s13063-023-07090-w)

Appendix 1. Model Informed Consent Form


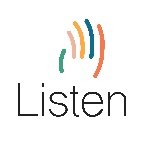


**Long Covid Personalised Self-managemenT support co-design and EvaluatioN (LISTEN)**

**Informed Consent Form**

**Participant Number**

**Please initial boxes:**

| 1. I confirm that I have read and understood the Patient Information Sheet for the LISTEN trial (LISTEN PIS Version …….. Date: …/…/……..). I have had the opportunity to consider the information and ask questions which have been answered to my satisfaction. |  |
| --- | --- |
| 1. I understand that my participation is voluntary and that I am free to withdraw at any time, without giving any reason, and understand that my medical care or legal rights will not be affected. |  |
| 1. I understand that data collected during the study may be looked at by individuals from Kingston University, Cardiff University, Swansea University, Bridges Self-management, UK regulatory authorities or NHS Trust staff, where it is relevant to my taking part in this research. I give permission for these individuals to have access to my information. |  |
| 1. I agree to my General Practitioner (GP) being contacted during my participation in the trial. |  |
| 1. I understand that the information collected about me will be used to support other research in the future, and may be shared anonymously with other researchers. |  |
| 1. I understand that my personal data, this consent form and the interview data I give will be stored on Cardiff University and Kingston University secure servers - accessible only to the LISTEN research team. |  |
| 1. I understand that any information that could identify me will be kept strictly confidential and that no personal information will be included in the study report or other publications. |  |

***Please turn over for page 2***

| 1. I understand that I may be assigned to the trial intervention arm and, in this case, that a trained practitioner will deliver the coaching sessions via a secure web video conferencing system or where required, by telephone. |  |
| --- | --- |
| 1. I agree to the coaching session(s) I have with the trained practitioner being audio recorded |  |
| 1. I agree to take part in the above study. |  |

**Please initial boxes:**

| **Optional:** I agree to be contacted for long term follow up for this study |  |
| --- | --- |
| **Optional:** I agree to be contacted to participate in an interview with a LISTEN trial team member.  I understand that the interview will be audio-recorded and sent for professional transcription |  |
| **Optional:** I give permission for some of my anonymised quotations to be used in future studies and to be shared with other researchers/organisations (both non-commercial and/or commercial). |  |

........................................................................... .......................... ........................................................

**Name of Participant Date Signature**

............................................................................ .......................... ........................................................

**Name of Person Taking Consent Date Signature**

Appendix 2. Model Participant Information Sheet

**
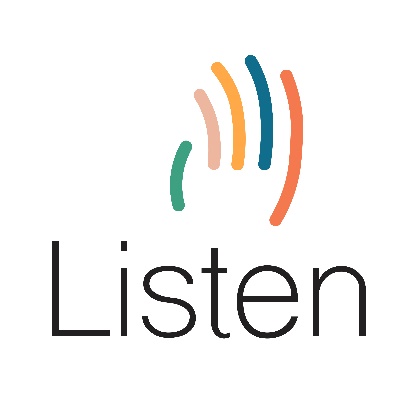
**

**PARTICIPANT INFORMATION SHEET**

**Title of the study:** Long Covid Personalised Self-managemenT support co-design and EvaluatioN (LISTEN)

We would like to invite you to participate in the LISTEN trial which has been set up by the Universities of Cardiff and Kingston University & St Georges University of London in collaboration with Bridges Self-Management, Swansea University, Kings College London and Lincoln University. The trial is sponsored by Kingston University. Throughout this information sheet, the use of the term “we” refers to the LISTEN trial team and sponsor.

Before you decide whether to participate, it is important for you to understand why the study is being conducted and what is involved. Please take the time to read the following information carefully and discuss it with others if you wish.

**What is the study about?**

Individuals with long Covid experience a wide variety of ongoing problems which can include tiredness and difficulty with everyday tasks and means they can struggle to return to their former lives. This is then made worse by uncertainty and a lack of understanding by some healthcare professionals.

The LISTEN team has worked with individuals living with long Covid to design a personalised support package including one to one support sessions and useful resources, to help others living with long Covid to self-manage their condition. The LISTEN trial will evaluate this new intervention compared to the usual care available in the NHS. We want to know whether this new intervention can help individuals with long Covid cope with the challenges they experience in everyday life.

We want to identify 558 people with long Covid to take part in the trial. The trial will use a randomised design. By randomised, we mean that if you agree to take part you will receive either usual NHS care or the LISTEN self-management intervention and the choice of group is based entirely on chance. We will use a computer system to decide whether you receive the new intervention or continue with the usual care that is available in the NHS.

**Why have I been invited and am I eligible?**

You are receiving this information as you may have reported some symptoms of Long Covid and told your GP/nurse/another health practitioner that you are happy to be told about the LISTEN research trial or you may have heard about the trial from another source and registered your interest via the LISTEN expression of interest online form.

You are eligible to take part in the study if you:

1. Have experienced persistent illness (at least one long Covid symptom for 12 weeks or longer) <https://www.nhs.uk/conditions/coronavirus-covid-19/long-term-effects-of-coronavirus-long-covid/>

AND meet any one of the following criteria:

- Positive Covid-19 test
- Positive Covid-19 anti-body test at any time point in the absence of Covid-19 vaccination history
- Loss of sense of smell or taste during the acute phase in the absence of any other identified cause
- Symptoms consistent with Covid-19 infection during the acute phase and high prevalence of Covid-19 at time and location of onset
- At least one symptom of Covid-19 during the acute phase AND close contact of a confirmed case of Covid-19 around the time of onset.

1. You should be aged 18 years or above and an English or Welsh speaker who has access to someone who can act as a translator

You are not eligible to take part in the study if you:

1. Have other health conditions which are progressive and palliative
2. You have been hospitalised for treatment of Covid-19 symptoms
3. Are currently taking part in another Covid-19 clinical trial with an intervention intended to help manage your long Covid symptoms

You can take part in the LISTEN trial if you have not yet accessed NHS long Covid services, are currently on the waiting list for NHS long Covid services or if you have already received usual care for long Covid but have ongoing symptoms.

**What does taking part in the study involve?**

If you are interested in taking part, you will be asked to fill out an expression of interest form and screening questionnaire online to check if you are eligible to take part in the trial (self-assessment). If needed, the research team can help you complete these questions over the telephone. When completed, your eligibility form may be selected for manual review. This means a member of the research team may telephone you to talk about your answers and confirm your responses.

Before you can be included in the study we will need to check that there is a LISTEN site open in your area. A list of open sites will be available for you to see on the expression of interest form. We will let you know by email if there is not currently a LISTEN site within your area.

If you are eligible for this trial and want to participate in the trial, you will asked to complete an informed consent form via a link sent to you by email.

You will then be asked to complete a number of questionnaires about your long Covid symptoms and how you are coping with them. These questionnaires will be emailed to you from the LISTEN trial. You do not need to complete the questions all at once. You can take breaks as needed .We will specifically ask you for:

1. Your name, age, contact details, including postcode so we can make sure that you are connected to a local research team
2. Information about your Covid (and long Covid) history
3. Information about how you manage day-to-day and your use of health and social care services.

If you are unable to access a computer/ internet, we can send you a paper copy of the quesionnaires in the post and a team member will be available to complete the questionnaires over the telephone with you.

Once you have completed the questionnaires, you will be randomly allocated to either the LISTEN intervention or usual care by a computer system. You will either be contacted by a practitioner and offered the LISTEN intervention for 3 months or you will continue with your usual NHS care that is available in the area where you live for 3 months.

If you are offered the LISTEN intervention, you will receive up to 6, one-to-one sessions with a healthcare practitioner through video call or by phone. These sessions will take place over a 10-week period to help you self-manage your long Covid. You will also have access to resources developed to help your self-management, such as a resource book and online help. The resource book will be sent to you in the post by the Bridges Self-management team.

After 6 weeks, we will ask you to complete two of the baseline questionnaires again to let us know what healthcare services you have used and your quality of life. You will be able to download a paper diary (optional) from the LISTEN website that you can use to help you to keep a record of your appointments.

After 3 months, we will ask you to fill out the some of the initially filled out questionnaires again. We expect that filling out those forms will take roughly 1 hr and 15 minutes to complete on each occasion. You do not need to complete the questions all at once. You can take breaks as needed .

If during the 3 months when you are participating in the trial (1) you are admitted to hospital for any reason, (2) your Long COVID symptoms get substantially worse or (3) there is any other incident you feel relevant that you haven’t previously reported to us, please inform us by using the ‘Report a Problem’ form available in your participant area on the LISTEN website. If there are any concerns about your health your GP will be informed.

At the end of the trial, we may ask you about your experiences of being in the trial. You might be invited to be interviewed by a researcher individually or in a group (focus group) using an online platform or telephone depending on what is most convenient for you. We would like to audio-record the interviews, and will ask your permission before the interview starts. If you have received the LISTEN intervention, then we may also ask to fill out some additional forms which will help us to assess whether the intervention is acceptable, feasible and appropriate for managing long Covid.

The interviews will take about 30 min to 1 hour – some may be shorter, some longer (but this depends on you/ group). You may be asked to maintain an online diary to take notes on what happens during the trial and how you feel about taking part in this trial.

The researchers would like to be able to use your data for future research and would like to have your permission to contact you in the future about further research. In the consent form you will be asked if you are happy for your data to be used in future studies and for us to contact you about other research opportunties

**Are there any benefits for me in joining the study?**

At this stage of the research, the direct health benefits for you are unknown, but by being involved, you will help us to gather evidence about different methods that may help individuals living with long Covid.

**Are there any risks for me in joining the study?**

There is a chance that you might find some topics sensitive or upsetting while taking part in the one-to-one support sessions or while answering the questionnaires. If you are participating in a focus group discussion, there is a risk that you might disagree with others. If disagreements occur, the interviewer will invite you both to put your point of view forward. Expressing different points of view for many people is a normal part of discussion and sharing of experience but for some people, disagreements can feel awkward or hurtful.

If you do experience any serious adverse effects while participating in the LISTEN trial, you will be asked to inform the study team and your clinical practitioner. You will be shown how to communicate this during your first one-to-one online session with your clinical practitioner.

**What if I become pregnant while in the study?**

If you are pregnant or become pregnant during the trial period you can continue to take part.

**Do I have to take part?**

No. It is your choice. If you are willing to take part in this trial, you will be asked to complete a consent form. If you do decide to take part but change your mind, you can withdraw from the trial at any time, without giving a reason. Not taking part, or deciding to stop, will have no impact or influence on your healthcare.

If you choose to take part and consent, the trial team will send a letter to your GP to let them know you are taking part in the study and what that involves.

**Withdrawing from the study**

If you participate and then decide that you want to withdraw from the trial, the information we have recorded about you whilst you were on the trial may still be used unless you request for your data to be destroyed. Please note that in some circumstances, it will not be possible to destroy your data for example, if the analysis has already been conducted or if reports/ publications have been written. All safety reports will also need to be retained.

If you lose capacity to be able to continue to consent to taking part in the study, you will be withdrawn from any further participation in the study and no further data will be collected. Data collected while you were able to consent would be kept and used in the study.

**How will we use information about you?**

We will need to use information from you for this research project. This information will include your name, contact details (including your postcode) and GP surgery.  People will use this information to do the research or to check your records to make sure that the research is being done properly.

People who do not need to know who you are will not be able to see your name or contact details. Your data will have a code number instead. We will keep all information about you safe and secure.

Once we have finished the study, we will keep some of the data so we can check the results. We will write our reports in a way that no-one can work out that you took part in the study.

### **What are your choices about how your information is used?**

- You can stop being part of the study at any time, without giving a reason, but we will keep information about you that we already have.
- We need to manage your records in specific ways for the research to be reliable. This means that we won’t be able to let you see or change the data we hold about you.
- If you agree to take part in this study, you will have the option to take part in future research using your data saved from this study.

### **Where can you find out more about how your information is used?**

You can find out more about how we use your information:

- at [www.hra.nhs.uk/information-about-patients/](https://www.hra.nhs.uk/information-about-patients/)
- by asking one of the research team
- by sending an email to [listen@cardiff.ac.uk](mailto:listen@cardiff.ac.uk)

All information will be kept private, confidential and secure. Your research data will be stored on secure servers in Cardiff, Kingston and Swansea Universities. If the results of the trial are published your identity will remain confidential. What you say/ communicate in the one-to-one sessions and interviews will be typed out by a professional transcriber to make a transcript. All identifiable information in the transcript will then be anonymised by changing your name and locations for example.

Your contact details will be kept securely on Cardiff University secure servers and/or in lockage storage facilities at Cardiff University.

The interview transcripts will be kept securely on Kingston University’s secure servers.

Only people involved in this trial or in making sure it is run correctly will have access to your information. No identifying information will be used in any reports.

**Who is organising and funding the study?**

The trial is being sponsored by Kingston and St George’s Joint Faculty Health, Social Care and Education, London (Surrey) and Cardiff University is managing the trial on a day-to-day basis. The trial is funded by the National Institute of Health Research.

**Who has approved the study?**

The trial has approval for conduct in the NHS from a Research Ethics Committee (REC) that is legally “recognised” by the United Kingdom Ethics Committee Authority for review and approval.

**Who do I contact if I have any concerns?**

If you have a concern about any aspect of this study, you should ask to speak to the trial researchers who will do their best to answer your questions [LISTEN@cardiff.ac.uk].

If you remain unhappy and wish to complain formally, you can do this by contacting

Professor Andy Kent

Dean of the Faculty of Health, Social Care and Education, Pro Vice-Chancellor

c/o EA to Professor Andy Kent, Executive Dean & Mary Hennessy, Head of Resources & Planning

Room KH0031, Kenry House, Kingston Hill, KT2 7LB

Telephone: 07585 729925

Email: a.kent@sgul.kingston.ac.uk

In the event that something does go wrong, and you are harmed during the research and this is due to someone's negligence then you may have grounds for legal action for compensation against Kingston University but you may have to pay your legal costs. The normal NHS complaints mechanisms will still be available to you (if appropriate).

**What happens next?**

If you feel you might like to take part in this trial, you can complete the online expression of interest and eligibility screening form on the LISTEN website, which will inform you if you are eligible to take part in the trial. If you are eligible, you will then be asked if you wish to consent to take part in the LISTEN trial and the trial team will check if there is a LISTEN site open in your area. You may get in touch with the trial team by phone or email (see contact details below) for more information if needed.

Thank you very much for considering taking part in this trial.

**The LISTEN Team Contact Details**


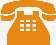
 Ring us on: 02922 514080 Monday to Friday (09:00 – 17:00)


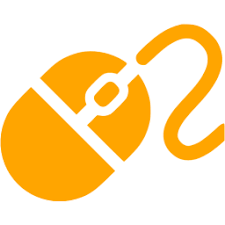
 Email us at: [LISTEN@cardiff.ac.uk](mailto:LISTEN@cardiff.ac.uk)


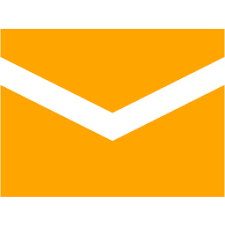
 LISTEN Trial Team, Centre for Trials Research

Cardiff University

4th Floor, Neuadd Meirionnydd

Heath Park

Cardiff

CF14 4YS


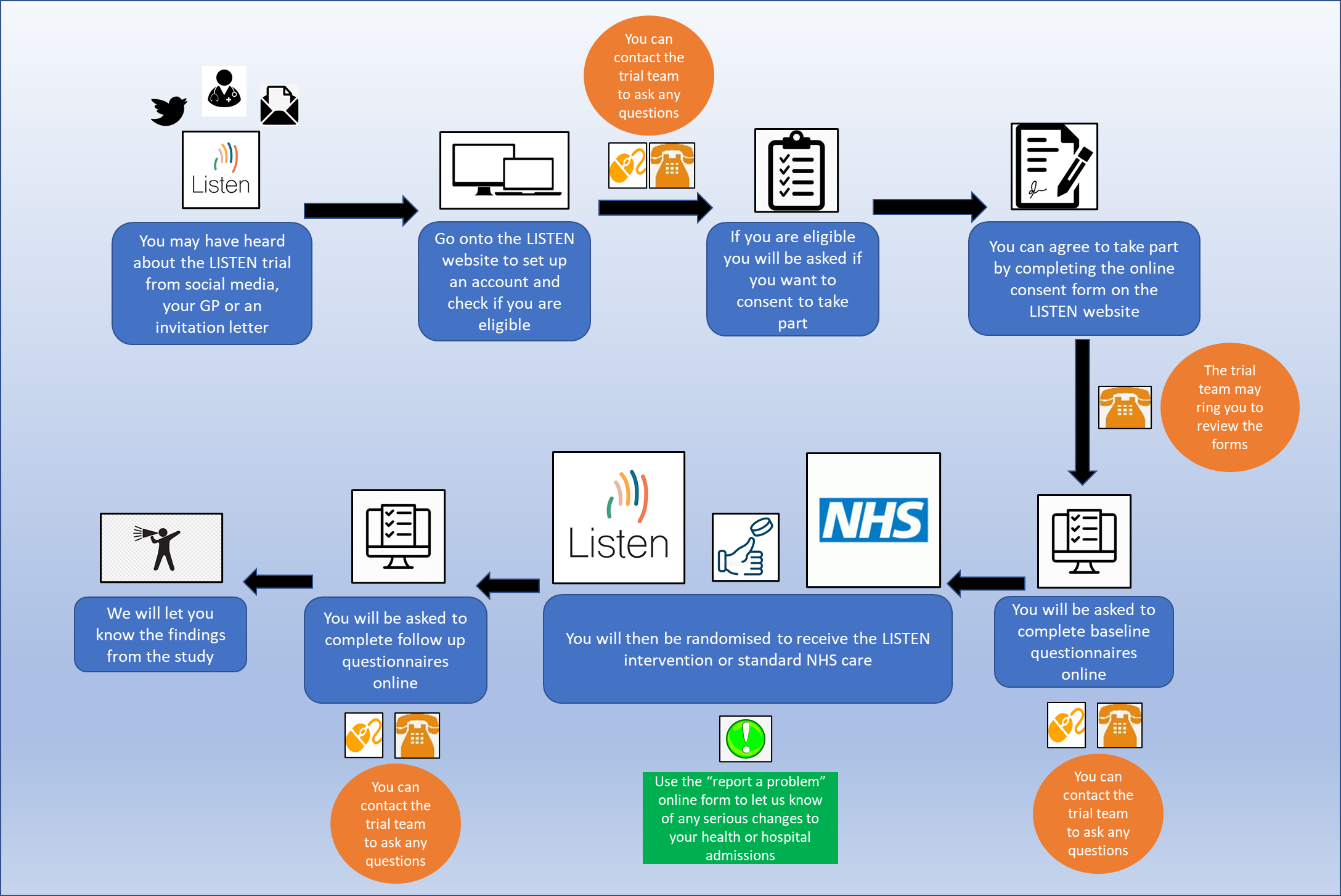

Supplement: Supplementary file 1 — Additional file 1: Appendix 1. Model Informed Consent Form. Appendix 2. Model Participant Information Sheet. [file 13063_2023_7090_MOESM1_ESM.docx]
